# Cyberbullying Characteristics and Prevention—What Can We Learn from Narratives Provided by Adolescents and Their Teachers?

**DOI:** 10.3390/ijerph191811589

**Published:** 2022-09-14

**Authors:** Jacek Pyżalski, Piotr Plichta, Anna Szuster, Julia Barlińska

**Affiliations:** 1 Faculty of Educational Studies, Adam Mickiewicz University of Poznań, 60-568 Poznań, Poland; 2 Institute of Pedagogy, Faculty of Historical and Pedagogical Sciences, University of Wrocław, 50-139 Wrocław, Poland; 3 Faculty of Psychology, University of Warsaw, 00-183 Warsaw, Poland

**Keywords:** cyberbullying, school bullying, qualitative research, prevention, school teachers, adolescents, empathy

## Abstract

The purpose of this article is to present the results of the study on the specific aspects of cyberbullying and prevention measures viewed from both the students’ and teachers’ perspectives. Cyberbullying is a severe threat to the individual and social well-being of young people. For this reason, it is important to understand how they perceive the phenomenon of cyberbullying, how they identify its causes, what they think about support, and the preventive measures offered through the lens of their own cyberbullying experiences. The study was conducted in a qualitative research paradigm. Students (N = 55) aged 13–16 from 25 junior high schools located in different regions of (blinded for the review) who had experienced cyberbullying incidents as victims, perpetrators, or bystanders, and their teachers (N = 45) were interviewed. They provided in-depth answers regarding cyberbullying incidents they had experienced and presented their attitudes and interpretations concerning those cases. The raw data were analysed by competent judges who defined a posteriori important categories that were useful for understanding the psychosocial mechanisms of cyberbullying and important dimensions of its prevention. The results proved a clear connection between participation in offline and online peer violence. The analysis of the statements showed that public/private types of cyberbullying involve different psychological and social mechanisms. Our findings confirm the importance of empathy as the buffering factor in cyberbullying perpetration. In addition, the limitations and inadequacy of the support and interventions offered by adults in cyberbullying cases have been emphasised in teens’ testimonies. The results may constitute grounds for formulating recommendations on the prevention of cyberbullying in the school context, taking into account the perspective of all actors involved.

## 1. Introduction

The aim of the current study is to present how young people and teachers perceive the phenomenon of cyberbullying from the perspective of different actors. We also focus on experienced causes, content and consequences, and perception of prevention and intervention measures utilised in schools.

Cyberbullying is most often defined as “any behaviour performed through electronic or digital media by individuals or groups that repeatedly communicates hostile or aggressive messages intended to inflict harm or discomfort on others” [1]. This covers a variety of different private and public methods like texts, videos, and theft of online identity that may have a different victimisation potential. Both online and offline bullying (without ICT involvement) has been associated with serious mental health problems in all involved actors—perpetrators, bystanders, but particularly victims who commonly suffer from depressive symptoms, psychosomatic problems, anxiety in the long term, and lower school achievements [2,3]. Bullying can lead to severe symptoms, such as self-harm and suicidality [4], which may even present in adults who experienced bullying while young [2,3], making cyberbullying a serious threat to both individual and social wellbeing. For this reason, it is important to understand how adolescents perceive the phenomenon of cyberbullying, how they identify its causes, what they think about support, and the preventive measures offered through the lens of their own cyberbullying experiences. The complexity of the phenomenon of peer violence requires the use of diversified research methods, comprising various theoretical perspectives and methodological traditions. Further, the respondent groups should be diversified and include adolescents, parents, teachers, and other school staff. Such an approach is an important standard recognised by the Society of Prevention Research (SPR) [5]. Particularly teachers form an important group of respondents since their knowledge and decisions influence the practice of prevention and intervention measures [6]. At the same time their incompetence and mistakes in this area may worsen the consequences of cyberbullying incidents.

Large research demonstrates the link between high-quality teacher–student relationships and numerous beneficial student outcomes such as reduced problem behaviour, increased academic achievement, enhanced school engagement, and improved social standing among peers. Synthesis of the literature on school-based interventions that included an outcome measure of teacher–student relationship quality revealed four approaches targeting teacher–student relationship quality by (a) increasing closeness; (b) decreasing conflict; (c) promoting social-emotional learning; and (d) emphasising relationship-driven classroom management [7].

Over the past fifteen years, a number of programs have focused on the prevention and education related to various forms of bullying. The crucial issue is whether these preventive measures are appropriate for the phenomenon of cyberbullying and meet the teenagers’ needs [8,9].

### 1.1. Key Knowledge Gaps in the Research on the Effectiveness of Anti-Cyberbullying Programs Addressed to Adolescents

The effectiveness of anti-cyberbullying programs has not been sufficiently assessed [10,11,12], with few studies demonstrating convincingly which methods are effective and for whom specifically. Additionally, little is known about the critical components of such programs that influence their effectiveness. It is also desirable for particular preventive actions to be part of carefully designed programs following the salutogenic model, in which prevention measures and enhancing participants’ empathic responsiveness is prioritised over combatting violence [13,14].

This line of thought is supported mainly by correlational studies that the cognitive and affective components of empathy have been shown to reduce aggressive behaviour [15,16,17]. Cyberbullies have also been found to have lower cognitive dispositional empathy towards their potential victims [18]. Few experimental research confirms the following directional relationship: affective and cognitive empathy activation significantly lowers the number of cyber-violence supporting acts by witnesses [14].

Some proposed intervention approaches are more general, with no specific provisions for cyberbullying in their prevention efforts. Among the proponents of this holistic approach are Olweus [13], as well as Espelage and Hong [19], who emphasise the need to integrate interventions aimed at traditional violence with countermeasures that target cyberbullying. Although school bullying has been explored thoroughly from the mid-1970s until the present, still little consensus regarding the relations between the two concepts has been achieved. Mostly the issues of validity of traditional bullying criteria are discussed by scientists, for example, the issue of repetition as in cyberbullying where the individual act is sometimes very harmful, as well as intentionality as it is easier to harm someone unintentionally online. For some scholars, those reasoning should lead to treating cyberbullying as a completely different phenomenon than bullying [1,20,21,22]. Some recent reports on programmes’ efficacy have indicated that dealing with only one of these phenomena on its own may paradoxically lead to increased violence [23]. However, other approaches have emphasised the need to take into account the particular characteristics of the digital media as well as specific cyberbullying mechanisms in the methodology of preventive measures, which should, accordingly, be tailored to the specifics of cyberbullying [24,25,26]. Programmes that address this second aspect tend to focus more on highlighting interventions aimed at specific roles in bullying following the triadic approach, such as mobilising bystanders [14,27,28,29].

Cyberbystanders are understood as students who “[notice] a social situation occurring among a cyberbully and a cybervictim” [30] (p. 124). It has been proved that cyberbystanders are the most numerous party engaged in cyber-bullying acts (20–55%) [30], comparing with 20–23% in the case of cyber-preparation and cyber-victimisation [31]; they are powerful social influence in creating both negative behavioral models—reinforcing the bullying and positive ones with responses such as intervention in cyberbullying cases [14,32,33,34]. Based on this approach, a successful programme should engage cyberbystanders and mobilise their willingness to provide informal help to their peers [14,30,34,35,36]. Data clearly states that the most effective factor in achieving this goal is the level of empathy in potential helpers [14,35,36,37]. Peers support along with the exploration of the role of empathy as the activator of cyberbystanders’ prosocial behaviour towards victims [14] enhance the development of behaviours aimed at coping with this form of violence.

Another under-researched area is the assessment of defensive actions undertaken by adolescents themselves. A couple of directions can be identified: the effectiveness of specific coping strategies for victims of cyberbullying, peer support and its effectiveness in diminishing the negative impact of violence on a victim’s wellbeing, as well as the exploration of the role of empathy as the activator of cyberbystanders’ prosocial behaviour towards victims [14]. Bystanders’ support for cybervictims has proved to be an important strategy in reducing cyberbullying [14,32,33,34]. Based on this approach, a successful programme should engage bystanders and mobilise their willingness to provide informal help to their peers [14,30,34,35,36]. Data clearly states that the most effective factor in achieving this goal is the level of empathy in potential helpers [14,35,36,37]. The possibilities for natural activation of empathy in an online context are particularly limited, due to both cyberspace conditions and the developmental specifics of adolescence.

To sum up, the main issues that are important in creating effective cyberbullying prevention projects are the cyberbullying criteria (especially intentionality, since numerous hostile acts online are conducted without an intention to harm), and the need for a more in-depth exploration of the key factors that moderate cyberbullying via addressing wider groups of participants and using a wider portfolio of research methods. The most important challenge is the need for controlling the impact of specifics of adolescent behaviour and its online manifestation in cyberbullying.

### 1.2. Psychological Aspects of Cyberbullying: The Impact of the Specifics of Adolescence, Online Communication, Empathy and Appearance Online

The period of adolescence is characterised by powerful egocentrism with its accompanying sensitivity about oneself, as well as conformism towards the peer group. These qualities make adolescents particularly amenable to social influence [38]. The peer group is the point of reference for creating one’s self-image, as well as satisfying basic social needs of belonging and affiliation [38]. The developmental specifics of adolescence are also characterised by an imbalance between cognitive abilities and motivation to control and anticipate one’s behaviour, which takes into account social expectations only to a limited degree. This is why adolescents often demonstrate poor impulse control combined with emotional impulsivity. The contact via digital media has the potential to increase this emotional impulsivity, causing teenagers to engage in behaviours they would not normally exhibit offline [39].

The defining element of cyber violence lies with its “digital context” which acquires the form of digital space and tools. Their impact goes far beyond the digital nature and takes on a psychological meaning manifested in the impact on neuronal processes [40] and the dynamics and quality of cognitive processes (attention, control, memory) [41,42,43]. At the same time, cyber-bullying participants remain under the influence of events, experiences and also peer norms or other phenomena embedded in the offline context. Such overlapping of the two realities and, depending on the approach, the mutual influence of various elements of either of such realities is verified, e.g., in the computer-mediated communication model [44,45] or social information processing” theory and Parasocial and Online Social Relationships Theory [46].

#### 1.2.1. Studies on Psychological Aspect of Cyberbullying

Some research has shown the impact of digital tools and networked environment on how information is processed [41]. Their use leads to alternating attention and increased superficiality of information processing [40,42]. Moreover, the mediated character of online communication creates a sense of anonymity and deindividuation, which limits the sense of responsibility by generating a cockpit effect [47,48]. In addition, certain aspects of computer software and how online messages are constructed, as well as information overload, collectively marginalise reflective processes, intensifying impulsiveness and multi-tasking [49,50]. This limits the influence of the subject on the course of cognitive processes and the evaluation of results.

The ease of activating automatic adjustment [42,50] manifests itself in the use of the most accessible stimulus: physical appearance, especially in relation to peer violence. Physical appearance is the first and the most accessible attribute in interpersonal contacts [51]. It plays a special role in “forming impressions” about others [52] and is a distinctive aspect of social perception, especially in childhood [53]. People tend to see in it the symptoms of one’s predilections to certain behaviours. In addition, presumptions concerning one’s character or personality features are inferred from one’s appearance [54]. Finally, physicality is the basis of stereotypes [51]. Physical appearance traits are easily used (especially by adolescents) as the basis for stigmatisation that may lead to harassment [55].

Results of multiple studies show significance of online image in the context of cyberbullying. The online image is one of the targets of attacks of cyberbullies, who in particular focus on the aspects of physical appearance. Sharing photos or video content presenting the victim in unfavourable light has significant victimogenic potential [35]. Forwarding compromising photos is seen by young people as the most serious anti-social behavior [56,57]. Some researchers even claim that this form of bullying has a more negative impact on the victim than peer violence used in direct contact [58].

Data indicate the significance of psychological competences for involvement in cyberbullying: empathy is negatively correlated with cyberbullying [18] and with a lack of emotional control experienced by cyberbullying victims and perpetrators alike [59]. The research findings show that inducing various actions which activate empathy in the triad—perpetrator, victim, bystander—limits the scope of aggression experienced by children at school [60]. The role of empathy as a mechanism modifying the attitude of bystanders towards victims is also confirmed by the research outcome showing that children equipped with a lower capacity for empathic reacting and pro-social involvement [61,62,63] are rarely prone to support victims. A similar relationship was found with regard to electronic peer violence [64]. Experimental studies also demonstrate that situational empathy activation has an impact on adolescent decision whether to forward or refuse to forward peer-ridiculing messages. In the conditions of the arousal of both affective and cognitive empathy, a significant decrease in the frequency of forwarding an abusing message has been found [14].

Mediated interactions generate deficits in empathic arousal. Tools and the digital space marginalise reflexive functioning and intensify automatisms. To analyse peer cyberbullying from the perspective of participants in these interactions, taking into account their perception of psychological competences (empathy) and the status of physical appearance is crucial if we are to effectively address the problem of cyberbullying.

#### 1.2.2. Current Study

The goal of exploring the specific aspects of cyberbullying and prevention measures viewed from both the students’ and teachers’ perspectives led to the research questions regarding subjectively perceived determinants of peer cyber-bullying and factors contributing to its escalation:

1. What forms of cyberbullying appear in adolescent narratives?
2. How are roles in traditional bullying and cyberbullying related?
3. To what extent appearance issues are a cause of cyberbullying?
4. To what extent is perpetration of cyberbullying intentional?
5. What role play the aspects of online and offline environment in cyberbullying engagement?
6. What is the role of bystanders of cyberbullying in adolescent narratives?
7. Is a level of selected psychological competence (e.g., empathy) associated with perpetration of cyberbullying?
8. From teenagers’ perspective, what is the role of teachers in the process of cyberbullying prevention?
9. What methodology of cyberbullying prevention is postulated by the participants?

## 2. Materials and Methods

A non-experimental study in the qualitative paradigm was planned. The semi-structured interview method was used. The study adopted a qualitative methodology to explore cyberbullying from the contextual perspective of various actors involved in the process (both students and teachers) [65,66]. Qualitative research design offers the opportunity to investigate bullying and peer aggression from the perspective of human interactions in a certain setting (e.g., school) and its cultural context [61]. Conversations and interviews create an opportunity to share one’s experiences with others, especially through constructing one’s own narrative [67]. Also worth mentioning is the usefulness of information obtained in this manner for the development and implementation of effective prevention and intervention strategies. Another advantage of the qualitative methodology, especially important in the context of adolescents assessed in the study, is its indirect character, which may lead to more open and sincere answers. In addition, the formal, linguistic aspect of the statements (available in qualitative methods) is a unique source of knowledge about how adolescents verbalise and structure their cyberbullying experiences—information which is not available through forms of measurement.

We aimed to cover all of the outlined topics during the semi-structured interviews, not in a predetermined order but rather following the flow of respondents’ narratives. The interviewers adopted a non-evaluative attitude and refrained from judgments and interpretations during the interview to assure safety and openness of the respondents who described their experiences concerning highly sensitive topics.

### Participants and Procedure

The research participants were students (N = 55; 30 boys and 25 girls) aged 13–16 from 25 junior high schools across different regions of (blinded for the review) who had experienced cyberbullying acts as victims, perpetrators, or bystanders. The selection of schools was based on the following criteria to assure variance of the sample:

- Size of town (schools from larger agglomerations: (blinded for the review), smaller cities: (blinded for the review), as well as suburban villages near to: (blinded for the review), and from the (blinded for the review);
- Area of (blinded for the review) (central, southern, eastern);
- Type of school (public vs. private junior high schools).

Two or three students were interviewed at each school. First, the headmaster was contacted and asked about cases of cyberbullying that had taken place in the two years preceding the study. The headmaster was then asked to check whether the parents of the students and the students themselves who had participated in the event would be willing to provide information to the researchers. In the event of initial consent, the researchers contacted the students’ parents/guardians and provided them with the necessary information about the study. After obtaining the parents’ consent, the researchers contacted the students asking for consent and arranging the date for the interview.

From the same schools, a convenience sample of teachers who had been involved in interventions in cyberbullying situations was selected (N = 45). The recruitment procedure was similar to the one for students. The teachers were also contacted after prior approval by the school headmaster.

Interviews were conducted from October 2015 to January 2016. The duration of the interviews ranged from 15 min to 1 h, depending on the willingness and readiness of the respondents.

The ethical procedure of the study included the following elements:

1. Consent of the Ethical Committee of the Faculty of Psychology at the University of (blinded for the review).
2. Consent of the parent(s)/guardian(s) in the case of students.
3. Voluntary participation and possibility to withdraw from the survey at any time.
4. Saving and publishing research material in a way that protects the anonymity of respondents.
5. Leaving the choice of method of documentation to the respondents (audio recording or written record).

Interviews were recorded and then transcribed, or their content was written down (depending on the respondent’s choice in this respect). Then the researchers read the responses and introduced categories for specific issues for analysis. The interviews were part of the Project (blinded for the review).

## 3. Results

The transcripts were analysed using thematic analysis rooted in the constructivist paradigm [68]. This approach consists in identifying, analysing, and reporting patterns (themes) noted in the collected research materials. It is described as a method that allows for effectively identifying, ordering, and connecting diverse threads in rich, unorganised databases through a five-stage approach: compiling, disassembling, reassembling, interpreting, and concluding [69]. To answer the nine research questions, the analysis was carried out in the aforementioned stages by members of an interdisciplinary research team acting as competent judges (different from those in the first stage). In the event of incompatible assessments the final decision was made following consultations between judges and was based on mutual agreement.

Please note: G14, 18—Girl 14 y.o., interview number 18; (B15, 33)—Boy 15 y.o., interview number 33); (WT, 30)—Woman, Teacher, interview number 30; (MT, 25)—Man. Teacher, interview number 25.

### 3.1. The Forms of Cyberbullying in Adolescent Narratives: Between the Private and the Public

Analysis of narratives about cyberbullying acts shows that young people use a great variety of forms and technical methods to bully others online. An in-depth analysis led to drawing the main typology of those acts based on the type of computer-mediated communication (CMC) adopted: one-to-one online communication (perpetrator(s) to a victim) and public online communication. Additionally, the form of outing leading to exclusion was a popular topic in teenagers’ narratives. Young people used instruments that allow one-to-one communication, where the exchange of communication is restricted to sender and recipient.

*The most unpleasant comments she sent me by private message on Facebook* (G15, 16),

*they wrote about her mother, that she had stolen something, etc. There were messages and pictures (…) sent privately* (G15, 24).

Contrary to popular belief that cyberbullying usually involves publicly disseminated material, here the material is only known to those directly involved. The one-to-one mode of communication mostly meant that in the majority of cases the perpetrators were not anonymous, since they were sending messages from their accounts on social networking sites and were easy to identify. On the other hand, some perpetrators used channels that make hostile messages or other content (pictures, films) public. For example, they used the public conversation mode of some sites, where more than one person can read messages or communicate publicly (e.g., on so-called “walls” of social networking sites).

*I have sent a modified picture of my friend in a group conversation* (B14, 40).

One of the victims described it this way:

*She posted sarcastic comments under my video on Facebook, such as: ‘How beautiful you are!’ or ‘Great idea!*’ (G15, 16),

*My friend posted a video. And I commented as a joke: ‘You bitch from school’. She deleted the comment* (B14, 45).

This distinction between private and public electronic peer violence acts is very useful from a conceptual perspective, since public mode adds new dimensions and mechanisms, primarily associated with the fact that other individuals (mostly students from the same peer group) join in the communication. Their involvement is usually described as adding additional unpleasant comments or backing up the initial perpetrators by “liking” their comments or disseminating the content further. This is particularly unpleasant for the victims when the material published is visual:

*I spotted it on Facebook. And one girl was quarrelling that she didn’t want this photo up. That was her in her underpants* (B15, 40), *they often take photos during breaks, after training, or when somebody sleeps and then they edit them. It’s unfair. I often try to cover my face when I sleep* (B14, 13).

There are cases when a perpetrator encourages other people to actively take part:

*One was publishing a post and wrote ‘Please leave a like if you don’t like him’. And then the others joined and also asked for likes. And then the whole thread started* (G13, 10).

In many cases, the public extended to people known from offline contexts, as the content was easy to access for others (e.g., friends of friends on social networking sites). This fact may make hostile acts potentially more harmful, since a victim may think that the content humiliating him/her is viewed and judged by a broader public and the “invisible audience”. In some cases, a victim has no access to actual content although he/she may be aware of the fact that others talk about him/her in a hostile way somewhere on the Internet:

*I have a friend—I don’t know what he is about… He never liked me and started to taint my reputation and send out various things on social networking sites. One of them was Ask.fm—and people started to change the way they saw me, and that was not true…* (G15, 17).

There are also forms of cyberbullying that skirt the borderline between public and private. The ones most often reported in interviews were identity theft and exclusion (ostracism). The first of these two is a cyberbullying act when someone takes over the account of the victim (e.g., stealing a password) and initiates on behalf of him/her private conversations or posts comments on social networking sites. These exchanges of messages are usually hostile towards other people, who erroneously think that they are sent by the victim. That means the reputation of the victim is endangered, since it is sometimes difficult to explain what actually happened and that a victim was not guilty. One drastic situation of this kind, when the content had been published openly, was described by one of the teachers:

*One of my students forgot to log out from Facebook. The other student spotted this and started to publish stuff. (…) The content was sexual; it was not pleasant for anybody. It was about him changing his sexual orientation to a homosexual one* (WT, 21).

In this particular case, the content was not only shared with other students but also the family of the victim, who alerted teachers at school about the situation. Another hybridised cyberbullying form of public and private electronic violence may take the form of exclusion:

*She was banned from the class’s Facebook group. Then the girls started a new group. Everybody from the class was invited except her* (MT, 7).

It is worth emphasising that in these particular cases perpetrators often used numerous technical channels and private and public ways of communicating harassment to a victim. However, in most cases one of the channels was dominant while others played a complementary role.

### 3.2. Offline Events as Causes of Cyberbullying

It follows from the analysis of the material that offline situations, i.e., real-life, face-to-face interactions are a source of cyberbullying. They are the source of conflict which is then transferred online, where it develops further. This phenomenon is exemplified by the response of one teacher: *The conflict had been going on before; it didn’t start on the Internet* (WT, 12). It sometimes happens that the perceived sources of cyberbullying are also rooted in the past. They concern previous experiences with peers that are implicitly reactivated in the context of a new stage of education. *That was unfinished business from primary school* (G14, 15). At times, violence starts online and is then transferred into the offline environment. *They laughed at X during a group conversation but X didn’t know about it. Then it spread into the real world* (B14, 20).

The responses suggest a variety of causes of bullying. They can be mundane, trivial quarrels and misunderstandings at school that are later picked up and continued in the form of posts on the Internet: *(…) two girls had a fight at school and it turned into a war, and they continued with abusive messages on Facebook* (B14, 6). The Internet is used as a forum to publicly harass through the way peers talk to one another in real world: *We were posting pictures of how she was addressing us, e.g., “Dumb cooze”* (G14, 14). Additionally, mutual hostility that escalates on the Internet where comments of people involved in various situations are posted: *Basically, we never really liked each other. There was a book from the library that she’d checked out (…) the teacher reminded her a year later that she hadn’t returned it, she said she’d given it to me [the book—authors’ note] to return it, and I hadn’t. And then, when I was at the library, the teacher said the book was found, that she returned it* (G14, 10).

Some acts of cyberbullying are amplified by parents who intervene by informing teachers about the negative experiences of their children. These situations result in comments among peers that constitute cyberbullying: *(…) on the same day that my mom went to school to sort out the issue with the book, those [aggressive—authors’ note] questions were posted. The language was typical for that girl* (G14, 10).

A recurring category of offline events that generate cyberbullying is that of direct interpersonal conflict. It becomes the source of negative emotions like jealousy, taking offense, providing motivation for behaviour aimed at humiliating, ridiculing, or taunting the target.

*They found out that she, that friend of theirs, had other friends. Because she told them she also had other friends. (…) they couldn’t accept it* (G14, 2).

*(…) she showed screenshots of chats when they were calling me names. My friend also admitted that (…) she was badmouthing me because she was feeling offended* (G13, 5).

Conflicts and resentment arising in offline situations may also provoke more violent forms of cyberbullying, specifically online hate speech.

*I have two friends outside of school, they don’t like each other, they used to be friends, but one of them went nuts (…) and got into drugs, smoking, drinking (…). And they were hating on one another on Facebook, calling each other this and that. One would post that you are this and that and drop a like if you hate him, the other wrote that if you don’t like the first one, like my post and commenting started, a whole conversation, and then it got to the point where they stopped talking to each other and they broke all contact* (B14, 6).

The participants pointed to offline conflicts and discord as sources of online aggression. The issues were often typical for this age group, involving quarrels over a girl/boy, jealousy, betrayal, taking offence, and seeking support from adults. The blending between the digital and the offline worlds is perceived and experienced, and the interaction between the two produces circumstances that trigger various symptoms of cyberbullying. This is clearly shown in a statement by one of the students: *Your life on the Internet affects your personal life, here at school and outside of school…* (G14, 14). This is also confirmed in the responses of teachers:

*This conflict had been going on before; it didn’t start on the Internet. (…) then the next step was on the Internet (…)* (WT, 12).

*Sometimes they fight in real life and it spills over. And sometimes the fight starts on the Internet* (MT, 3).

*Perhaps the fact that aggression has moved to the Internet is the reason research shows reduced aggression, and we as teachers have noticed a similar trend, but it is simply going on elsewhere* (MT, 7).

### 3.3. Appearance-Based Cyberbullying

Physical appearance traits easily dominate perceptions, thus giving a pretext for negative comments. This is well captured by one of the students’ statements: *Different in some ways, often in appearance* (B13, 11). Any deviation from so-called normal facial features is often immediately recognised and often becomes the object of nasty comments: *(…) these are the kids from the inclusive classes, (…) they are typically on the receiving end of taunts because for them they are different, so this is the person to laugh at, make jokes about, that difference* (WT, 20), or vulgar comments on physical attributes: *Fat, greasy pig, dumb blonde* (B15, 9).

Adversarial comments may also use embarrassing photographs, often purposefully distorted or modified.

*He took a picture during class. It looked like I was picking my nose. He immediately posted it to Facebook. With a comment: ‘a pig in class’* (B15, 40),

*(…) a girl uploaded a photograph and someone modified it maliciously* (G13, 9),

*in primary school, they were also photoshopping my pictures, but they deleted them quickly (…) Here, they also often take pictures of one another during recess, after training, or when someone is sleeping, and then they modify them…* (B14, 13),

*someone doodled something nasty on someone else’s picture, modified it, or they added captions to pictures, saying you are this and that* (B14, 13).

The same is true of physical limitations: *(…) the reason was short videos recorded by a girl who was excused from PE; she was a rather vicious person who was simply recording her friends struggling during PE. The girl who was being recorded (….) was particularly awkward and later a clip [was posted—authors’ note] showing how she is straining, trying to jump over the vaulting horse, doing other stuff* (WT, 3), or untypical behaviour, *She (the victim) is strange. Different or something. She behaves like… (unable to explain)* (G14, 30), which becomes the reason for bullying.

### 3.4. Intentionality and Cyberbullying

Perpetration of cyberbullying, in the testimonials of Polish teenagers, rarely takes the form of intentional activity. Many bullies described their actions as impulsive, undertaken without planning, and with no intention to hurt another student. The following two accounts illustrate this point: *Seriously, no, the other person did not take it seriously, because I wasn’t being serious. I called someone a “moron”, but later I started laughing about it with my friend and we started calling each other “moron” just for fun* (G14, 20). *There are these funny situations and you just post something with an inappropriate picture, on purpose, taunting someone, for example, about their name* (B15, 18). Teachers express similar opinions, saying that online aggression for adolescents is often *a form of entertainment* (WT, 10) and *joking* (MT, 24).

Moreover, aggressive behaviour is often justified by teenagers as having been provoked by some stimulus, e.g., a communicative act by their victim on the Internet. *They started calling our classmate names (online) because that guy from our class was calling them something, (…) someone said he was this and that, the other started calling him names and another printed what the other one wrote and complained to the teacher, he was summoned, so he came to school acting all sorry, thinking he was the victim* (G 13, 27). In addition, young people say that this type of scenario often results in the escalation of the conflict. *He was her ex-boyfriend (…) it was just out of anger because he broke up with her or something and so it happened/friends of that girl went after that boy, so the friends of that boy went after the friends of that girl for picking on him, and it was like this vicious circle* (B15, 26). When describing the vicious circle, teachers mention that, with each retaliation, *the students are more and more cruel* (WT, 23).

Another frequently mentioned feature of cyberbullying among peers is the inclination to blame the victim: *If she wasn’t keeping to herself so much, it would have been different* (G14, 26); *She shouldn’t have told her mom, who made a big deal out of it* (B14, 45). The teenagers in our interviews have a way of talking about victims that appears to ignore their emotional perspective: *She (the victim) is strange. Different or something. She behaves like…* (G14, 30). Teachers’ statements also identify the students’ distant attitude towards the victim: *It’s difficult to get through to that person (victim)* (WT, 23), sometimes echoed by the teaching staff: *You cannot help someone against their will* (MT, 24).

Bullies often struggle to explain the motivation behind their actions. When asked about it, some answered *I don’t know, I just did it* (B14, 44) mainly due to distancing and avoiding. Such phenomena may result from moral disengagement, a situation-based cognitive reconstruction that leads to positive or neutral attributions of moral transgression and violence approval, which is confirmed by other participants, i.e., student bystanders who are only describing what they saw: *(they do) the first thing that comes to mind, without regard for consequences* (G16, 33), and school teachers/counsellors/psychologists: *I don’t think they realised it was hurtful (…) I’d say in most cases it is the failure to realise, not just that they are breaking the law, but not realising how they are hurting someone else (MT, 7).*

Cyberperpetrators often become aware of potential consequences for the victim only when he or she talks about them directly or when someone else (e.g., the parent of the student who has been the target of cyberbullying) brings up this issue: *While I was having ‘fun’ with others (co-perpetrators), I wasn’t feeling sorry for her. But then I started feeling sorry for her* (G14, 26). In some cases, bullies fail to appreciate the harm they have caused even after some time has passed (even despite clear evidence). *But there was no fighting and no one got hurt* (B14, 45). When that happens, an apology tends to be forced, artificial, and rarely leads to actual improvements in peer relations: *They said they were sorry, but they were afraid. They said sorry, but it was forced because they got scared and only then deleted all the comments, but by then there were screenshots* (G14, 44); *I apologised to her on Facebook, making it look as if it were my idea, so as not to get into more trouble (…) It was better to do it on the Internet* (B14, 45).

### 3.5. Online Environment and Social Influence as the Sources of Cyberbullying

The digital environment and electronic devices create specific conditions that induce adolescents to engage in negative and inappropriate behaviour. The sense of anonymity offered by the Internet facilitates actions that violate social norms. *(…) there they could feel, they thought they would be anonymous, that they would get away with it* (WT, 12) or as a student notes *it is simply easier to write certain things than to say them to someone’s face* (G14, 4). *Writing on the Internet is a massacre; it’s spreading* (G14, 6). It also creates opportunities for those who have little chance to express aggression in real life: *Shy people are more likely to talk back on the Internet than in real life* (B14, 24).

Teachers also point to digital communication as conducive to the brutalisation of peer relations: *but discussion forums are also the place where vulgarisms appear and students are offended. The thing is we do not know who the person we are dealing with really is.* (WT, 2). Technological advances offered by the Internet carry a potential for harming others: *(…) in my opinion the fashion for showing one’s pictures and reporting on almost every minute of one’s life and competing for the number of “likes” is a tremendous threat* (WT, 14). The teacher also stresses the negative role of social networks: *(…) first and foremost all those social networks that cause so much harm to children (MT, 2).*

On the other hand, reports provide material in support of the overpowering authority of the peer group for engaging in reinforcement or perpetration of cyberbullying. The commonness of a given behaviour within a group institutes a kind of social norm that serves as a standard for behaviour. Criticism is limited, and the dominant attitudes are conformism, as well as impulsive reproduction of the behaviour within the peer group. *We’re all doing it for fun and no one takes offense... It didn’t occur to me at all that he could be angry about it. I got pranked like that as well, and maybe it felt a little uncomfortable, but when I saw everybody was doing it, I relaxed and made no fuss about it* (B14, 9).

*Some people said I was exaggerating because everybody was doing it and I was the only one to take offence* (B14, 18).

*(…) I don’t know how she could take offence, all my friends were surprised* (B14, 24).

*When girls and boys started reminiscing about it all, I was really upset; I didn’t know what to do… I was crying* (G14, 15).

*There is peer pressure* (WT, 23).

What is more, the victims’ reports suggest that even if the expressions of cyberbullying are privately renounced by adolescents, it has little impact on their behaviour. Despite the sense of unease associated with hurting others, they replicate that behaviour for fear of being criticised by the group and for the need for social approval and affiliation. *Some were saying that, for a long time, they had been feeling uncomfortable when pictures and comments were directed at them, but they didn’t say anything, because nobody did. And so it started to look like everybody was having fun* (B14, 9).

*I don’t intervene in any way or else I’ll get blamed as soon as I have a problem myself* (B13, 1).

The mechanism of generating such a group is often underpinned by fear and group exclusion anxiety. It is revealed in a statement by one of the teachers: *One person likes tormenting others. Other girls joined in so as not to get on her wrong side. (…) They are more powerful as a group, they feel strong* (WT, 30)

Cyberviolence can also be triggered by a group that is established to fulfil this very aim, i.e., doing harm (perceived, obviously, as having fun). The consequences of the operation of such groups are subjectively experienced by the victim as particularly distressing. *They set up this group [on Facebook—authors’ note] and humiliated me on that group together with others. I felt horrible. I was psychologically vulnerable and every insult was getting to me. So when I read all those comments on that group and all that, let me tell you, I didn’t feel like going to school, I just didn’t want to be seen* (G14, 15).

Distancing oneself from a close-knit peer group is sometimes seen as a violation of unspoken rules and often gets punished online. This is illustrated by a girl’s statement, which reveals reasons for cyberbullying: *If she wasn’t keeping to herself…* (G14, 24). Groups also punish members for breaking the rule of not notifying adults about peer behaviour: *We knew she was talking (with adults about the matter). She should have talked to us* (G14, 14). This aspect of social pressure is captured perfectly in the following statement by a teacher: *I think there is great fear of being the same as the person targeted, they stick to that one way or another, taking the side of those acting cool and seen as funny* (WT, 1).

### 3.6. The Role of Bystanders in Cyberbullying Dynamics

The statements of young people in our qualitative research also suggested that the actions of bystanders are of key importance in the dynamics of online aggression: *Some people support them, others post comments for them to stop and calm down, and there is a third group that sees everything but does not respond* (B14, 4)*; Everyone picks the side they want to be on, I have my friends, she has hers, they will obviously be on her side, and my friends on mine. And then there are the bystanders, those who are, shall we say, not associated with either of us, I heard they were making fun of her for taking offense for this sort of thing* (G14, 19). In the accounts of teachers, it is not the victim but teenage bystanders who are the most likely to intervene when someone is targeted online: *Witnesses, more often than victims, come with screenshots and files on pen drives. It’s a measure of our success that children know what to do. This is thanks to teachers’ training* (WT, 24)*; In most cases, we find out from other students* (MT, 25). The role of bystanders can have a prosocial impact, as in the following account, where they actively defend the victim: *When someone was criticising some post because it was poorly written, I defended the person that was criticised* (B15, 6).

However, passive behaviour is a much more common response: *When I saw that, I just ignored it, because I didn’t want to make it worse for her by taking part* (B13, 1)*; I don’t intervene in any way, or else I’ll get blamed as soon as I have a problem myself* (B15, 2). Defending might be perceived as risky, since the bystanders have to challenge popular, powerful bullies—which requires both skill and courage. This is apparent in the following account by a young girl: *These girls that were gossiping online are seen as dominant, they get even, they ridicule others. Nobody responds for fear of retaliation* (G14, 20). Thus, it may seem more reasonable and safer to remain neutral as a cyberbystander and to avoid the company of low-status victims, or even to support the bullies. This behaviour can be also interpreted via the social influence and peer pressure mechanisms confirming the complexity of mechanisms underlying online teenage bullying. As teenagers observe and monitor each other’s reactions to cyberbullying episodes, they might conclude that a lack of defensive actions is because the majority approve of what goes on. Young people we interviewed stated that for a bystander to intervene takes strength and courage: *In primary school, I had this classmate who was in his world and others were making fun of him and taking pictures of him. I reacted by saying that it was idiotic to take pictures of someone against their will only because they were acting strange. These images were never uploaded, by the way* (G13, 9)*,* as well as specific values and model behaviours that may solve the problem not only for the victim but sometimes also save the bully from the painful consequences of his or her actions, as is illustrated by the following statements: *Some people have their heads on straight and they can help. And they did, and so I wrote to that person and apologised, and it all turned out well* (B15, 5)*; I started having second thoughts when older guys made me realize what I had done. That I’m younger, and I’m the one who’s mocking* (G15, 25).

When they do take action, bystanders in cyberbullying cases collected in our research were more likely to join the bullies and reinforce the victimisation rather than help the victim. Bystanders’ active participation in the victim’s persecution can manifest both offline: *I was in trouble both online and at school. The girls in other classes who were reading the comments would whisper to one another when they passed me. They didn’t do anything to me, just whispered* (G14, 44)*,* and online: *It was funny. I usually joined in (on Facebook)* (G14, 26). The actions often take the form of harming the victim thoughtlessly and reflexively: *I mean, you don’t think what you “like”, you just give likes to everything? In general, I click like on everything. Why shouldn’t I?* (G13, 10). Teachers also emphasised the role of group mechanisms that may intensify thoughtless antisocial actions of cyberbystanders: *There is peer pressure. When confronted individually, they seem to realise [what they are doing—authors’ note]* (WT, 23). This corresponds with a well-established mechanism, which is also true for offline bullying. In both contexts group norms concerning bullying and peer group, hierarchical structures influence bystanders’ willingness to defend the victim.

Another important feature of cyberbullying that emerged in the interviews was role overlap. This overlap, or even evolution within a given role, seems particularly pronounced in the case of cyberbystanders, and is to a large extent a consequence of role ambiguity. The unique character of online environment and the way people function in cyberspace means that, unlike in direct interactions, boundaries between roles are blurred, which young people describe as follows: *I don’t know, I try to avoid those situations and not get involved because sometimes I like both people and I don’t know whose side to take. And sometimes it’s hard to be objective* (B14, 3). A good illustration of role evolution typical for bystanders is the story of an individual who started as a bystander adding to the cybervictimisation of the victim (*reinforcer*), then offered support to the victim and reported the case (*defender*), but in legal proceedings was eventually declared to be an accomplice: *This picture was sent to me as well, and I forwarded it to one other girl. I sent it to her, and she went all in, because she sent it to everyone, even to people who didn’t know her. I mean, this is no excuse, because I also did something wrong by passing it on (…) and this is why we have to appear in court soon. … (Then) I started feeling sorry for her, so many people have seen it and laughed at her (…) I wanted to help her so it wouldn’t get worse and so people would no longer mock her, but the school counsellor talked to them and said they were to stop it and start helping that girl* (G, 13, 9).

The transition towards prosocial behaviours in the accounts of teenagers contains themes of affection, empathy, and regret: *I started feeling sorry for her, so many people have seen it and laughed at her (…) It was funny at the time, but part of me wanted to make sure more people wouldn’t see it, so I told the teacher. After all, I like her* (G13, 9). The teenagers also made references to schoolmates’ solidarity and positive peer norms that disapprove of violence. *Maybe not in online comments, but in private they did, my friends. To show their solidarity in this conflict, they recorded a similar video and posted it just for laughs on their profiles, to show that they too could imitate the same as me. This was a positive response. They were very supportive of those two close girlfriends of mine* (G14, 19). With respect to generating positive, prosocial norms of behaviour, i.e., responding to violence, teachers emphasise the importance of modelling such behaviours among peers, which helps overcome the pervasive indifference towards acts of cyberbullying: *All it takes is writing that you are not cool with what is going on and that your opinion is different. It’s like opening a door so that the next person finds it easier* (WT, 4).

### 3.7. Lack of Particular Psychological Competences as a Source of Cyberbullying

Deficits in adolescents in terms of empathically responding to a peer’s discomfort are often mentioned in the accounts of teachers and labelled in a variety of ways, e.g., as lack of awareness: *(…) they didn’t realise (…)* (MT, 9)*, they don’t do it maliciously (…) they do it as a joke* (MT, 10), *I think it usually results from lack of awareness, but not only of the fact that they could be breaking the law, but the lack of understanding how one person can hurt another* (MT, 9)*,* cruelty, *Young people are cruel* (WT, 14), or joking, *for him it was a good joke* (WT, 12)*, these youngsters treated it as a joke* (WT, 14)*, It’s a form of entertainment* (WT, 15), *Yes, it was a picture and what can I say… You know, it was an opportunity to have some fun, so I did* (G15, 12). A telling symptom is that even personal, negative experiences of victimisation fail to engender positive, empathic responses: *I felt really stupid, you know, how I could have behaved that way, having been treated like that by others, knowing how the other person must be feeling. It was just this reflexive action* (G15, 12). In other words, one of the key learning mechanisms of cognitive empathy, namely, one’s own experience can be inhibited in the digital environment.

The suffering of others is often overlooked, unnoticed, and, most importantly, not anticipated. Even external intervention containing a message about the state of the other person does not later arouse empathy, but fear focusing on the Self: *They said they were sorry, but they were afraid. The apology was forced because they got scared (…)* (G14, 24). *I apologised to her on Facebook as if it were my idea, to make it go away, it was better [to apologise—authors’ note] on the Internet* (B14, 24).

The reports by teachers point to egocentric projection as the basis for anticipating the states of others: *for him, it was a joke, he didn’t see it as hurting his friend (…) he thought his friend would be laughing as well. That’s how it was. He saw nothing wrong with it* (WT, 2). Taking a group’s standards as the reference point favours a reinterpretation of bullying: *It didn’t occur to me at all that he could be angry about it. They had pranked me like that as well, and maybe it felt a little uncomfortable, but when I saw everybody doing it, I relaxed and made no fuss about it* (B14, 9). *Friends were saying ‘how could someone take offense at that’* (B14, 24). This justifies negative behaviour and inhibits empathy as a response.

Perpetrators’ statements showed inadequate perception of their behaviour as benign. They tend to think that their actions bring no harm to others: *I mean, that’s why you send those jokes. It’s like a game, a conversation; you wonder what the other person comes up with. It’s like a competition, but mainly just a joke* (B14, 9).

The categories used in descriptions of other people’s discomfort are vague and unspecific: *she wasn’t feeling great, it wasn’t fun for him* (B14, 9) *or I don’t think he felt cool (B14, 8), (…) he was not doing well* (G14, 15). A teacher’s statement confirms this interpretation: *I think they can’t call it* (WT, 13).

The analysis of language used by adolescents indicates difficulties in verbalising the affective states of peers (emotions or moods). This seems to be an important factor inhibiting the activation of reflective (cognitive) empathy with its underlying concept code. The problem with naming peers’ affective states is due no to ignorance of these categories but rather to their selective availability. There is a clear asymmetry between the descriptions of perpetrators and victims. Victims verbalise their affective states precisely and appropriately: *I felt sad that he humiliated me in this way, (…) also ashamed—after all, someone was sort of fiddling with my picture, used it and modified it just for laughs (…) then I was simply mad, I think (…)* (B14, 9), *I was really upset, I didn’t know what to do… I was crying* (G14, 14)*; For me, it was mostly about health, it was really stressful (…) I generally tend to get sick from time to time, and when he started all that, it got worse and I had terrible headaches, almost to the point of fainting* (G15, 8). The perpetrators’ reports are unspecific, general, and inadequate. The difference in perspective is hardly surprising, given what we know about egocentrism in adolescence.

In some cases, cyberbullying gets reinterpreted and becomes transformed: *But there was no fighting and no one got hurt* (G14, 24). This statement clearly shows that the consequences of offline physical violence are directly available and therefore have greater regulatory potential. Online violence lacks that spectacular expression, making its symptoms more difficult to spot and more susceptible to reinterpretation.

The analysis of teachers’ statements confirms deficits in control and behavioural inhibition: *And when they get angry, they either start fighting and brawling, or they take the phone and write to one another. On Messenger I think* (B14, 6); *Recently, there’s been a fight, and it also started on Facebook, because the boys put each other through the wringer so much that when they met in real life, fighting ensued. (…) Those three said they were provoking and they apologised, they should not have provoked and the other one shouldn’t have hit them or shouldn’t have let himself be provoked and we decided that was his weakness, he gets provoked, so when he is ready to get physical, he is to turn on the spot and come to me or his friend* (MT, 3).

The statements of adolescents indirectly suggest that their behaviour online is typified by the lack of thought about its consequences: *It hadn’t occurred to me what someone else could feel* (B14, 9). Impulsivity and reactivity are manifested by the widespread use of vulgarisms: *I saw her post the video and this jester’s nature awoke in me, and so I wrote ‘you cunt’ to her* (B14, 24). Negative, vulgar expressions dominate communication (…) *Your face is like a vomited, expired squirrel cutlet; Why weren’t you at school you prick; I can’t bear to look at your mug, you must be a faggot* (WT, 6). Impulsivity and lack of control are also manifested in problems with clear and logical verbalisation of thoughts, causal formulation of narration. They include large numbers of interjections and digressions, confounding the argument and detracting from its communicative value. The reports indicate the dominant role of automatic regulation, deficits in attention control, and routine and repetitive behaviour: *I, for one, am in this group on Facebook called “Fame Seekers,” and there (…) we like each other’s photographs. I don’t even look at them, just click like, like, like immediately* (G13, 5).

### 3.8. The Role of Teachers in the Process of Cyberbullying Prevention

The material collected includes a number of thematic threads pertaining not only to acts against violence but also to education and prevention. *The school counsellor always helps us (…) There were talks with the counsellor and she persuaded us to stop bullying this girl [i.e., the victim—authors’ note] and to accept her instead, and this was a breakthrough, at the beginning it seemed funny, so we were sharing it, and only then most people understood that it may not be cool or funny* (G13, 9).

An important factor in preventing situations potentially leading to peer violence and providing solutions to them is a proactive approach by the teacher, which may involve the use of social media, not only as a platform for conveying instructional materials but primarily as a tool for promoting educational goals and building social bonds with young people. *I’m on Facebook as a school counsellor. I keep this account just for my students, next to my private account. (…) They do write various things on their walls and being on Facebook allows me to pick up on what’s going on with them. So I contact them in private and write that I can see that they are sad, and I offer my help, to which they typically reply that they would like to come and talk* (WT, 21)*; I write to the sender that this post should not perhaps have appeared when I notice something on the school Facebook group* (MT, 7).

As the collected data indicate, the victims and witnesses of cyberbullying seek help from adults: the majority of respondents reported an act of cyberbullying to an adult person—a parent and/or teacher/school counsellor, especially if the act was highly victimising. *Those who come are mostly victims, sometimes also witnesses (…) these kids know that they can get help here* (MT, 7); *From the very beginning I kept my mum informed and we went to see the teacher together the next day* (G14, 44). The interviewees note a positive tendency to turn to adults for assistance: *It seems to me that students’ attitude towards teachers and parents has changed for the better. Now they tend to see adults as competent users of the Internet, who know what goes on there, and not like some aliens from a completely different world* (MT, 6). How adults are contacted by the victims and witnesses of cyberbullying depends on many factors and may not involve face-to-face interaction. *It depends on the circumstances on both sides. Those involved may do it directly or through their mates. Sometimes they put an anonymous note under your door saying that there is a problem and asking you to visit a website and see (…)* (WT, 21). The main criteria based on which adolescents choose the addressee of their request for help are trust and expectation of an adequate response (i.e., lack of exaggeration and being discreet). *A friend of mine went to see the school counsellor, who then called that guy [the perpetrator—authors’ note] and they simply had a conversation* (MT, 13).

As the material obtained in the study indicates, present-day educational goals can be achieved thanks to a significant presence of adults both on the Internet and offline, as evidenced by this testimony of a young person: *One of the coaches is my Facebook friend. He’s an active Facebooker (…) and president of the sports club, posting a lot of sports events and some info about the club (…)* (B15, 12). Even though such activities may not seem to be strictly connected with counteracting cyberbullying, they are a form of what is known as positive prevention, which relies on the enhancement of individuals’ resources. The relationship between an adult educator and a young person provides many opportunities to strengthen the potential of the latter, with the Internet being a convenient medium for prevention and with teachers availing themselves of the “bright side” of social media: *For young people, the Internet is a socialising space, a source of information and personality development. Internet activities are an alternative to ‘doing stupid things’* (G15, 8). The next statement by a school counsellor demonstrates the awareness that well-established bonds with young people should be perceived as an important protective factor: *When you have good contact with them, they indeed come (to talk about various problems)* (WT, 24).

Recognition of the Internet as a platform for building positive peer relationships in virtual communities, whether they are the sole form of contact between peers or complement an existing offline relationship, can be seen in this statement provided by a school employee: *I know and it is really interesting for me that kids establish such class groups with teachers. There’s a group admin and a vice-admin who post reminders about class tests. When someone has missed classes they ask others what the lesson was about, so this group is really helpful as a source of information about the current class and school affairs. Some teachers are members of such groups and others, who are not, may be informed by students about what is going on in the class social media* (WT, 16).

It is crucial for teachers to be aware of the fact that they have to identify students who are likely to become victims, bearing in mind that cyberbullying is often a mere extension of traditional forms of peer violence. *Victims are typically chosen from the group of pupils with a weak position in the class. They become victims of aggression; you don’t attack someone strong* (WT, 24). Some of the interviewees seem to notice opportunities in online activities typically seen as dangerous or morally dubious, such as online games: *playing games supplied by foreign servers, he had to learn the language and his English improved in writing and speech* (WT, 15). Another statement showing appreciation of the opportunities offered by the Internet for developing interests and training social skills is this: *(…) [The Internet –authors’ note] is a space, where—I can see that—even kids who seem to not have any hobbies, get interested in something. They come across videos showing people doing some things and they get inspired to develop some new interests. They are looking for information, they want to discover something about the world (…) after some searching they may find something they will start to identify with; (…) They work through everything that happened in the school, using their specific language, of course. Perhaps they are venting their anger and other emotions that arose in them in response to what happened in the school but they could not show their reaction in front of the teacher. This is very frequent in such groups* (WT, 16).

### 3.9. The Methodology of Cyberbullying Prevention—Perception, and Expectations

In the light of the collected material, both the preventative and interventional measures undertaken at school are often rooted in punitive, retributive philosophy. The following testimony corroborates this observation: *I think we’ve had one class [about cyberbullying—authors’ note] and we were threatened with the perspective of dealing with a prosecutor* (G14, 26)*; One student was told that he would get a low conduct mark but ultimately he got a good one; Each school has a statute based on education law and the only penalties stipulated in the statute are the teacher’s or head’s reprimand, so they were officially reprimanded. Because the situation took place in the spring, they got low conduct marks at the end of the year—if you get an official reprimand, the final conduct mark has to be the lowest on the scale. But students couldn’t care less. Penalties don’t work, their effect is zero* (WT, 12). Anti-cyberbullying actions are typically undertaken post factum, rather than as preventive measures: *Students were bullying another student on Facebook (…) The class teacher asked me if I could do something about it. So I said I’d devote a class to this problem* (MT, 7).

Another recurrent topic in the material under analysis was that of divergences between young people and adults concerning their respective assessment of frequency, appeal, and efficiency of prevention and intervention actions. *And the next day after the lesson the fake account of that student was deleted. So they got the message* (MT, 8)*; We did have such classes but not many. We wrote something, we talked, and played the game called “Mafia”. These activities were supposed to build a bond among us, but they didn’t change much. We surely won’t miss each other* (G14, 30). These statements, however, were produced in different situations and conclusions should be drawn with caution. Another testimony given by a student exemplifies the ambivalence in assessing the effectiveness of interventions undertaken at school: *In fact, little was done at school in my opinion, lowering the conduct mark didn’t have any effect because it was not on his certificate. His parents weren’t even notified; he only talked to a psychologist. Well, our relationship got better; he stopped doing what he used to do, so it really is better for me, but on the other hand…* (G15, 17).

One of the most important conclusions supported by the young people’s testimonies is that educational meetings about violence prevention are typically conducted in the form of lectures and they inspire little interest on the part of participants: *Not very interesting. The teacher just talked to us* (G14, 26)*; They could organise it differently. It’s mainly theory* (B14, 28). Furthermore, the content of such lectures is often considered to be abstract and quite far removed from reality: *We had classes on how to use the Internet in a safe way, conducted by some teachers. And they weren’t really interesting. They should talk about situations in which we can find ourselves and, for instance, how to solve problems. What we heard instead was that the Internet is something blah blah and that it cannot be deceived* (B14,16)*; we had lessons on cyberbullying too, both with people specially invited to our school and as part of the IT course, most basic stuff (…) It was a talk (…) generally we weren’t listening too closely (…) it was just obvious things, like if we notice someone is being abused we have to report it, but surely we don’t need to be told such obvious things …* (G13, 39). These statements point to a discrepancy in perspectives (of students and teachers) from which the prevention of cyberbullying is approached.

Ambivalence can also be found in the students’ evaluation of the usefulness of educational meetings on violence prevention: *The meetings are obligatory so we attend. Cool, classes are cancelled during those meetings* (B14, 28) but also: *We miss important classes, and then we are behind on the material* (G14, 30). The meetings are typically described as boring and largely ineffective: *These meetings are not particularly successful* (B14, 28); *It’s neither interesting nor encouraging* (G14, 30)*; There are boring programs—some people talked about something* (B14, 45). These statements illustrate the perceived passive character of efforts that make no use of young people’s activity and involvement. Efforts of this kind are not suitable for the existing challenges. Due to the fully controlled nature of this kind of address, a lecture is a “safe” form of delivery for the teacher. When the meetings are well designed and prepared, they do exert a real positive influence, as noted by this student: *[after a class on cyberbullying—author’s note] our understanding and acceptance increased (…) people no longer tease this girl but they are trying to help her* (G14, 20).

According to individual teachers, intervention measures are often graded (e.g., a reprimand first, then being summoned by the school headmaster, notifying parents, and finally informing external authorities), as this statement indicates: *if I have a perpetrator, I have to scare them off first, my aim is to make sure that what they did will not happen again. I don’t want to appeal to their emotions because it would take longer and may not have an immediate effect, but my method is effective—the perpetrator gets it at once. Further work may also be necessary, but most of the time the first step is sufficient* (MT, 7).

Many students participating in the study point out that effective cyberbullying prevention, or, more broadly, Internet threat prevention is necessary. More specifically, they suggested that cyberbullying prevention should be carried out through the same medium in which it occurs: *the Internet should be discussed on the Internet* (B14, 11). Interviewees expressed expectations related to the interactive character of the medium, its attractiveness, and motivational systems: *a lot of videos and rewards* (G15, 25). It is also necessary to recognise the varied character of young people’s educational needs, and the limitations of preventive actions carried out sporadically, as evidenced by this statement: *I know my peers and I know that such a film [about a woman being a victim to cyber aggression—authors’ note] won’t change their conduct on the Internet* (B13, 8). This might point towards a tendency in educational work to use stereotypical cases that do not stimulate reflection or can even promote habituation—a desensitisation to exposure to others’ discomfort.

Using narratives based on authentic experience in planning prevention can be conducive to its effectiveness. The need to have educational materials illustrated with examples is also reflected in the following statements: *It would be much better if those directly involved had a voice* (B14, 28); *Better give examples—it’s more interesting* (B14, 45). Young people’s suggestions include greater interactivity and a strong emotional context, as reflected in this statement: *More communication with the instructor. To encourage those who would like to take an active part but won’t volunteer* (G14, 29); *Avoid showing slides. Somebody should talk without shying away from graphic details* (G14, 30). Well-thought-out use of knowledge obtained from students—addressees of prevention measures—in designing anti-violence programs should contribute to their effectiveness. Being open to the addressees’ perspective helps in obtaining precious knowledge about whom they follow on social media and who is, therefore, an idol and authority figure for them: *Serafin is a Youtuber making funny videos that he posts as comments about the hits of the Internet. But recently he started posting more serious stuff, for example, about refugees or the school system reform* (G15, 16). Another advantage following from diagnosing young people’s needs regarding violence prevention is becoming familiar with specific topics and problems that can be taken up in school activities, such as debates or workshops: *It should contain a definition and deal with privacy issues, such as the degree of privacy on Facebook so that we can find out what others know about us and what can happen. Not everybody is aware that photos and posts they upload can be seen not only by their friends but also by strangers* (G14, 10); *I think that posting those silly videos should be restricted, so when a stranger decides to watch something like that, they would have to accept it, it’s just stupid and unnecessary. People have no sense of shame today and post anything; it didn’t use to be like that …* (G14, 2).

## 4. Discussion

The aim of our qualitative projects was to gain a more in-depth understanding of the phenomenon of peer cyberbullying: its features, conditions, and relations with the offline reality, from the perspective of young people involved in different roles in cyberbullying as well as their teachers. Our analyses confirmed the legitimacy of taking up the issues contained in the research questions.

Based on our results, we can conclude that both forms of cyberbullying via private identifiable means and public communication channels are used. This latter could be interpreted as an online version of indirect bullying, establishing negative attitudes against the victim and opening the door to other direct forms of bullying [70]. From a practical perspective, both identified forms of cyberbullying need to be addressed separately through effective prevention and intervention strategies. This is due to different underlying mechanisms and coping strategies that are based on different types of online communication. This is mainly connected to public dimension of some cyberbullying acts that made them more severe in terms of victimisation [71].

Offline situations as the cause of aggression exhibited on the internet seem to explain what teachers describe as declining aggression in direct contact. It would therefore seem that aggression has not diminished, but rather transformed, migrating into the digital space [72]. The opposite direction has been very rare—still we have found testimonials when young people situated origins of bullying online—e.g., in closed classroom fora.

Victims of bullying often go on to become bullies themselves, while perpetrators are victimised, with offline problems replacing online ones, and vice versa. Substantial evidence points to various links between offline and online bullying roles in terms of cyberperpetration as a predictor of traditional school bullying, traditional school bullying perpetration experience as a cyberperpetration predictor, and links between the roles (the bully–victim status) [30,73].

Generally, our findings help interpret the results of much quantitative research, which often finds an overlap between offline bullying and online bullying [21,74,75]. Effective prevention programs should therefore also acknowledge offline situations viewed as a potential source of cyber-bullying.

Our analyses confirm that social media is perceived and used by young people as a space for presenting visual aspects, including external appearance. With its open and interactive nature, social media is an ideal arena for self-presentation and publishing photos ridiculing others. Both forms of activity exploiting the external appearance fulfil the self-valorisation function and serve as a status generating tool in the Internet, with the latter being a particularly acute form of cyber-violence.

Many expressions of cyberbullying are aimed at the features of physical appearance. This is confirmed by the salience of physical features in social perception [51]. Appearance-related attributes are very “concrete” and stereotyped in their nature: they dominate perceptions and become an easy excuse for derisive comments. Additionally, our findings showcase the need to emphasise the topic of cautious online self-presentation in cyberbullying prevention programs. It remains equally important to emphasise a simplified character of inferring about a person’s traits based on his/her physical attributes [55]. Materials and exercises that reveal the superficiality and inadequacy of such a strategy may cause addressees to reflect upon their behaviour and re-examine reasons for cyber violence.

From the data collected in the interviews, we can conclude that both bullies and bystanders, whether adult or adolescent, tend to describe the perpetration of bullying as thoughtless, unintentional, and unpremeditated. These results provide evidence for the need to implement non-restrictive no-blame approaches for perpetrators in cyberbullying prevention and intervention actions. Other data clearly show that the dynamics of cyberbullying cases are dependent upon other unintentional factors, such as specific technological settings, friend vs. acquaintance of the cybervictim, the bully’s popularity, clear vs. unclear circumstances, perceived fairness of the behaviour of the involved parties, directness or proximity to the cybervictim, the severity of the act, etc., which influence the process and the outcomes of bullying acts [36,37]. Those mechanisms spark a tendency to blame the victim and adopt attributions that show emotional and rational distance. That was partially proved by results of other studies suggesting that students avoid victims, especially in classes with strong status hierarchies and imbalance of power, where friendship with victims increases the risk of loss of position and being victimised oneself, which makes the likelihood of intervention low, as bullies select victims with few or no friends [76].

In some cases, bullies fail to appreciate the harm they have caused even after some time has passed (even despite clear evidence). This stands in line with quantitative data on moral disengagement of cyberperpetrators [77], which is higher and much easier in the online context due to reduced social and contextual cues available [78].

The adolescents’ and teachers’ statements confirm that the specifics of communication mediated by digital tools create particular circumstances that facilitate behaviour that reinforces online aggression. The content of the narratives usually suggests that peer violence on the Internet is viewed not as a category of behaviour, but unrelated, isolated acts of violence, each of which has a unique context. The adolescents failed to draw more general conclusions, e.g., about the category of situations that trigger aggression. However, many accounts contain a more or less openly expressed intuition that the specifics of the online environment facilitate involvement in and reinforcement of bullying. These findings correspond with many previous reports pointing out that the sense of anonymity (sometimes present even in situations when a perpetrator is known to a victim) concerning both parties in the interaction is a key modifier of mediated communication [48,79]. Based on these results, enhancing teenagers’ knowledge on the impact of computer-mediated communication on their behaviour seems an important condition for an effective approach to reducing online aggression.

Another aspect of the analysis of collected material suggests that the peer group active online is perceived as the source of norms that regulate behaviour, which is especially apparent when computer mediated communication is used. Harmful online behaviour subordinated to peer norms increased victimisation. The universality of peer standards and fear of consequences of exclusion seem to justify and, at the same time, be the cause of negative actions on the Internet. This is why it is crucial to take into account the dynamics and significance of peer group in developing effective cyberbullying prevention programs. Particularly important is raising awareness concerning peer influence and the automatic and impulsive online behaviour that may be caused by it.

Our results highlight the importance of bystanders’ reactions to cyberbullying as a powerful social influence mechanism in creating positive anti-bullying behavioural models [14,30,36,80]. The collected findings on the role of bystanders stand in line with the knowledge of the bystander effect [81] when those who witness bullying shift responsibility for responding to it to the teacher or the victim’s friends [61]. Bullies are accepted according to pro-bullying class norms with certain strategies or unintentional actions, bystanders can reinforce bullies or support and defend their victims, both online and offline [82]. It underlines different factors and mechanisms operating in school class contexts that influence bystanders’ behaviour: adherence to peer group norms; perception of group hierarchy; homophily; pluralistic ignorance; bystander effect; social identity; and moral disengagement among adolescents, which might contribute to how bullying is perceived, shape responses to it, and explain why it is so pervasive [83].

Analysis of the statements indicates control and inhibition deficits. They increase the probability to engage in hostile online behaviour. The control functions, as well as empathy, are subject to changes through education programs. Therefore, it is of crucial importance for the prevention programs to include strategies directed at enhancing various manifestations of executive functions. These should include improvements to implementation plans and strategies, operational memory, and mindfulness training [84]. Additionally, the kind of preventive measures that include the element of induction appear to optimise empathy [85]. Cognitive empathy responses, especially role-taking, are shaped by environmental factors, i.e., parental and teachers influence and preventions programs. Modelling, the use of induction and perspective-taking are often mentioned as parenting techniques that facilitate the development of cognitive empathy [85,86]. Though we have not measured empathy levels, our interviews provided unique insights into the way participants perceive and interpret the suffering of others, and the responses it elicits. Furthermore, the method offers a glimpse of how costs and benefits are calculated with respect to responding to the apparent victimisation of peers, as well as the awareness of others’ perspective and ability to anticipate their emotional states.

Online mediated contact with those suffering reduces involvement of the basic mechanisms of automatic, affective empathy [86,87]. This is due to the lack of direct contact with a universal stimulus (e.g., facial expressions, tone of voice, gestures), which would automatically trigger aversive arousal [88]. In addition, advanced mechanisms responsible for triggering reflective, cognitive empathy [86,89] are impeded. On the one hand, this is the product of the characteristics of digital devices that reinforce superficiality and impulsivity; while on the other hand, the problem lies in the developmental specifics of adolescence: deficits in inhibition, control, and self-distancing. Numerous results confirm the importance of empathy in reducing cyberbullying both as a personality disposition [18] and a situationally activated factor [14,35]. This needs to be addressed in the process of creating effective online bullying preventive measures.

A key role for effective prevention and problem-solving is teachers’ commitment to building and strengthening traditional (offline) relationships among young people and supporting their online engagement. As the findings show, students seek help from adults with whom they have a good relationship and who appreciate the importance of new media in the lives of young people. This positive image should not however be generalised to teachers as a group, as it pertains to individual adults, especially psychologists and school counsellors, who are trusted by young people. Such individuals typically undertake actions, often informal ones, on their initiative rather than act within a general scheme developed at the institutional level.

A crucial component emerging from our research is that solving issues related to cyberbullying needs to be implemented within the “understanding approach” of the teachers. There is a need in developing diagnostic competency in recognising potentially problematic situations (e.g., the importance of identifying high-risk groups as an element of systematic action against peer violence [90].

The most important conclusion that can be drawn from the analysis of the material collected in our research is that a significant number of measures undertaken by educators draw on many different approaches and models rather than on a systematic program of peer violence prevention and intervention. This clearly shows the need for a teacher approach shaped by an empathetic, personalised understanding of adolescents’ needs and motives. This approach also requires openness and a non-judgmental attitude towards young people and their problems, both offline and online. Nonetheless, our data shows the opposite. The typical approach of teachers may be characterised as rather distanced and based more on punitive strategies than real interpersonal contacts. This may negatively impact the students’ willingness to turn to teachers as helpers and mediators during cyberbullying incidents. This negative tendency may be even reinforced by unattractive didactics of many educational meetings concerning online risks as stated by our young respondents.

In terms of their universal characteristics, the interventions given as models in the literature tend to be multifaceted and interdisciplinary [74,91]. As such, they involve integration of interactions addressed to individuals (e.g., victims), the whole class, and/or the whole school community, incorporating both regular and occasional activities, proactive as well as reactive, i.e., initiated on an as-needed basis. The interdisciplinary approach is defined as involving experts from various disciplines (educators and psychologists, but also IT specialists, lawyers, police officers, etc.). The programs themselves should have strong foundations in theory, empirical findings, and knowledge of best practices, with systematic assessments of their outcomes [5,9]. This approach to cyberbullying prevention has proved to be particularly effective [80].

Another apparent shortcoming of preventive measures seems to be educators’ lack of ability to move from an abstract, often oversimplified level of description of the phenomenon to particular strategies acknowledging the experience of young people. Pupils express preference for an entirely different approach in prevention (e.g., more interactive, engaging forms, more taking into account the context of new media). It is also worth mentioning the need expressed by young people to use in educational activities the personal stories of individuals involved in cyberbullying. This kind of approach is likely to appear more worthwhile to target groups, since stories told by peers are more engaging than purely theoretical information. Moreover, such stories are also more memorable and comprehensible, as pointed out by Kreuter, et al. [92]. The fact that the online environment is not only the source of the problem but also a medium through which it can be solved was noted by Vandebosch [11]. This means that its engaging character is a clearly important distinguishing feature of the Internet as a means for preventive actions. Deliberate use of this quality seems to be important in terms of making productive use of the Internet in this context.

## 5. Conclusions

We have explored the key influencing conditions in order to show the complexity of the phenomenon of cyberbullying as well as its dependence on various contextual and relational factors. Implementing these results into effective prevention and intervention measures, in particular educational settings, is a challenge.

One of the fundamental aspects is to create a prevention strategy that would be more sensitive to the perspective of individuals who have important and unique knowledge. Unfortunately, the question of how young people perceive the source of cyberbullying, efficacy, and appropriateness of prevention and education efforts is rarely asked. Anti-bullying programs developed and/or implemented by their addressees based on participatory research are few and far between [34,93,94,95], with even fewer that take into account the perspective of not only adults, i.e., teachers, but also that of the adolescents themselves [29,93,95,96]. On the other hand, the importance of this approach finds support in this article’s reported data as well as empirical findings that show that ignoring the needs of adolescents or implementing inadequate interventions when dealing with peer relations that involve cyberbullying discourages students from seeking adults’ help, thereby limiting the range of effective forms of support available in these difficult circumstances [66]. This is precisely why asking what we can learn from the young victims, bystanders, and perpetrators of cyberbullying are so important. Opinions of the addressees of these actions (satisfaction, needs, perceived outcomes) should constitute the foundation for creating new and improving existing support programs, as was done in the research presented here.

The study is a source of both recommendations for future studies on cyberbullying prevention as well as for effective preventive measure creation.

The specific prevention strategies we recommend based on our results are as follows:

1. Cyberbullying is not a monolithic phenomenon but rather a collection of qualitatively different sub-phenomena that should be adequately addressed when developing preventive measures. This as our results shows may be caused by differences in technology use (methods) but also social aspects such as connection to traditional bullying or public versus private mode of hostile attacks.
2. Cyberbullying prevention should include online communication mediated in private and public channels as well as offline problems of young people, particularly those connected to traditional bullying.
3. Cyberbullying prevention should address all three roles present in cyberbullying cases: victims, perpetrators, and bystanders, as all actors influence the dynamics of particular cases. Bystanders have an especially powerful social influence in creating positive anti-bullying behavioural models.
4. Cyberbullying prevention measures should be adopted from the individualised perspectives of young people and teachers, since attitudes of young people in this respect closely relate to actual behaviour (e.g., providing support or indifference while witnessing cybervictimisation)
5. Cyberbullying prevention should emphasise the topics that are universal for the stage of adolescence: cautious online self-presentation as well displaying manifestations of the superficiality of judgment based on looks; the significance of the peer group and its consequences.
6. Cyberbullying prevention should focus on enhancing teenagers’ knowledge on the impact of computer-mediated communication on their behaviour in cyberbullying actions.
7. Cyberbullying prevention should focus on developing in adults an understanding of the importance of new media in the life and education of adolescents and on supporting teachers in online engagement. Another crucial element is to sustain and deepen traditional (offline) relationships with young people in the new media environment, underlining the role of teachers in modelling antibullying standards.

Thus, the results of the study provide both educational recommendations and suggestions for future studies on cyberbullying prevention. By using a qualitative approach, we were able to better capture the context and perspective of adolescents and teachers. This ensured a more comprehensive understanding of the complexities of violence perpetrated with the use of new technologies from the perspective of victims, perpetrators, and bystanders.

The study naturally has its flaws and limitations. The first limitation of the gathered data is its local context. The study was conducted only with (blinded for the review) pupils and teachers so its generalisability to other countries is limited. Yet, the issue of generalisation of our findings seems more complex. Although the achieved results are not representative in a statistical sense, the idea of their generalisation seems to be justifiable. Based on the rules of sample selection criteria in qualitative studies the sample meets the requirement of homogeneity (our respondents were precisely selected with regard to the education stage) and internal differentiation (selection of schools from various urban and rural settings, public and private schools, schools equipped with various Internet infrastructure as well as various socio-economic backgrounds). Specificity of qualitative research permits finding generalisations as long as the research is aimed at seeking qualitative knowledge, i.e., that concerning the motives and mechanisms of cyber-violence, as well as relations, for example, those between a witness’s experiences and offering help to victims, or configurations of, for example, competences regarding empathy and control and the phenomenon of cyber-violence. Thus, their significance lies in facilitating the recognition and understanding of the respondents’ natural environment. They also serve as a prerequisite for generating hypotheses concepts as well as application requests. The repetitiveness of observations in subsequent interviews is, most certainly, a pivotal factor [97].

Second, it seems overly optimistic to expect that the presented qualitative material will provide a definite response as to what kind of preventive measures ought to be taken. Likewise, no clear-cut methods of compensating the deficit of competences conducive to violent behaviours can be inferred. Our interlocutors told us about their feelings, experiences, and behaviours, but it lies with the researcher, who also takes into account other data from this domain, to decide how the gathered material could be used to develop a preventive strategy.

Third, due to the complexity of the attitudes and behaviours of young people, as well as the experiences and events described by them, one ought to remain cautious about the provided straightforward responses. For example, the attractiveness of the Internet should not fully determine one’s decision to use it as an exclusive tool in education programs with the form of a lecture, considered as “dull”, being eliminated. There are advantages and deficits to both forms. For example, online conditions increase superficiality and rashness [41,42], whereas an interesting lecture may engage reflexivity. Everything depends on the adequateness and attraction of the content of the message—the choice of the medium seems much less important.

Fourth, our study is situated within a qualitative paradigm. Thus, we do not have the data that allows us to provide credible comparisons between different subgroups (e.g., boys and girls). This shortage is also connected to our sampling that is not representative for such comparisons. Nonetheless, our findings may be implemented in further quantitative research, particularly for defining variables and categories and constructing research tools.

Finally, the limitation of semi-structured interviews is the use by participants of subjective criteria to evaluate what they identify as the root cause of the phenomenon. Thus, it sometimes happens that they do not distinguish between the causes and symptoms of a phenomenon. Nevertheless, such material offers an opportunity to identify the subjective perspective of people directly involved in a given phenomenon.

## Data Availability

Not applicable. The respondents were informed that the raw data will not be published and available.

## References

[1] Tokunaga R.S. (2010). Following you home from school: A critical review and synthesis of research on cyberbullying victimization. Comput. Hum. Behav..

[2] Lereya S.T., Copeland W.E., Costello E.J., Wolke D. (2015). Adult mental health consequences of peer bullying and maltreatment in childhood: Two cohorts in two countries. Lancet Psychiatry.

[3] Vassallo S., Sanson A., Olsson C.A. (2014). 30 Years on: Some key insights from the Australian Temperament Project. Fam. Matters.

[4] Schneider S.K., O’Donnell L., Stueve A., Coulter R.W. (2012). Cyberbullying, school bullying, and psychological distress: A regional census of high school students. Am. J. Public Health.

[5] Gottfredson D.C., Cook T.D., Gardner F.E., Gorman-Smith D., Howe G.W., Sandler I.N., Zafft K.M. (2015). Standards of evidence for efficacy, effectiveness, and scale-up research in prevention science: Next generation. Prev. Sci..

[6] Nappa M.R., Palladino B.E., Nocentini A., Menesini E. (2021). Do the face-to-face actions of adults have an online impact? The effects of parent and teacher responses on cyberbullying among students. Eur. J. Dev. Psychol..

[7] Polling D.V., Van Loan C.L., Garwood J.D., Zhang S., Riddle D. (2022). Enhancing teacher-student relationship quality: A narrative review of school-based interventions. Educ. Res. Rev..

[8] Olweus D. (2005). A useful evaluation design, and effects of the Olweus bullying prevention program. Psychol. Crime Amp Law.

[9] Ttofi M.M., Farrington D.P. (2011). Effectiveness of school-based programs to reduce bullying: A systematic and meta-analytic review. J. Exp. Criminol..

[10] Della Cioppa V., O’Neil A., Craig W. (2015). Learning from traditional bullying interventions: A review of research on cyberbullying and best practice. Aggress. Violent Behav..

[11] Vandebosch H., Vandebosch H., Green L. (2019). Cyberbullying Prevention, Detection and Intervention. Narratives in Research and Interventions on Cyberbullying among Young People.

[12] Smith P.K., Vandebosch H., Green L. (2019). Research on Cyberbullying: Strengths and Limitations. Narratives in Research and Interventions on Cyberbullying among Young People.

[13] Ostaszewski K. (2014). Zachowania Ryzykowne Młodziezży W Perspektywie Mechanizmów Resilience.

[14] Barlińska J., Szuster A., Winiewski M. (2018). Cyberbullying Among Adolescent Bystanders: Role of Affective Versus Cognitive Empathy in Increasing Prosocial Cyberbystander Behavior. Front. Psychol..

[15] Caravita S.C., Di Blasio P., Salmivalli C. (2009). Unique and interactive effects of empathy and social status on involvement in bullying. Soc. Dev..

[16] Jolliffe D., Farrington D.P. (2006). Examining the relationship between low empathy and bullying. Aggress. Behav..

[17] Jolliffe D., Farrington D.P. (2011). Is low empathy related to bullying after controlling for individual and social background variables?. J. Adolesc..

[18] Steffgen G., König A., Pfetsch J., Melzer A. (2011). Are cyberbullies less empathic? adolescents’ cyberbullying behavior and empathic responsiveness. Cyberpsychol. Behav. Soc. Netw..

[19] Espelage D.L., Hong J.S. (2017). Cyberbullying prevention and intervention efforts: Current knowledge and Future Directions. Can. J. Psychiatry.

[20] Dooley J.J., Pyżalski J., Cross D. (2009). Cyberbullying versus face-to-face bullying. Z. Für Psychol./J. Psychol..

[21] Olweus D. (2012). Cyberbullying: An overrated phenomenon?. Eur. J. Dev. Psychol..

[22] Whittaker E., Kowalski R.M. (2015). Cyberbullying via Social Media. J. Sch. Violence.

[23] Dehue F., Völlink T., Gunther N., Jacobs N., Campbell M., Bauman S. (2018). Stop Online Bullies: The advantages and disadvantages of a standalone intervention. Reducing Cyberbullying in Schools: International Evidence-Based Best Practices.

[24] Ortega-Ruiz R., Del Rey R., Casas J.A. (2012). Knowing, Building and Living Together on Internet and Social Networks: The ConRed Cyberbullying Prevention Program. Int. J. Confl. Violence.

[25] Pieschl S., Porsch T., Kahl T., Klockenbusch R. (2013). Relevant dimensions of cyberbullying—results from two experimental studies. J. Appl. Dev. Psychol..

[26] Schultze-Krumbholz A., Schultze M., Zagorscak P., Wölfer R., Scheithauer H. (2016). Feeling cybervictims’ pain-the effect of empathy training on cyberbullying. Aggress. Behav..

[27] Menesini E., Nocetini A., Palladino B.E. (2012). Empowering Students Against Bullying and Cyberbullying: Evaluation of an Italian Peer-led Model. Int. J. Confl. Violence.

[28] Salmivalli C. (2010). Bullying and the Peer Group: A Review. Aggress. Violent Behav..

[29] Wójcik M., Mondry M. (2017). Student Action Research: Preventing bullying in Secondary School—Inkla Project. Action Res..

[30] Pfetsch J., Wright M.F. (2016). Who is who in Cyberbullying? Conceptual and Empirical Perspectives on Bystanders in Cyberbullying. A Social-Ecological Approach to Cyberbullying.

[31] Cook C.R., Williams K.R., Guerra N.G., Kim T.E., Jimerson S.R., Swearer S.M., Espelage D.L. (2010). Variability in the prevalence of bullying and victimization: A crossnational and methodological analysis. Handbook of Bullying in Schools: An International Perspective.

[32] Kozubal M., Szuster A., Barlińska J. (2019). Cyberbystanders, affective empathy and social norms. Studia Psychol..

[33] Szuster A., Barlińska J., Kozubal M., Wright M.F. (2016). In Search of a Simple Method: Is a Human Face an Effective, Automatic Filter Inhibiting Cyberbullying?. A Social-Ecological Approach to Cyberbullying.

[34] Wójcik M., Hełka A.M. (2018). Meeting the needs of Young Adolescents: Abbl anti-bullying program during Middle School Transition. Psychol. Rep..

[35] Barlińska J., Szuster A., Winiewski M. (2013). Cyberbullying among Adolescent Bystanders: Role of the Communication Medium, Form of Violence, and Empathy. J. Community Appl. Soc. Psychol..

[36] Machackova H., Dedkova L., Sevcikova A., Cerna A. (2016). Empathic responses by Cyberbystanders: The importance of proximity. J. Youth Stud..

[37] Machackova H., Dedkova L., Sevcikova A., Cerna A. (2018). Bystanders’ supportive and passive responses to Cyberaggression. J. Sch. Violence.

[38] Schaffer H.R. (1996). Social Development.

[39] Kowalski R.M., Agatston P.W., Limber S.P. (2012). Cyberbullying: Bullying in the Digital Age.

[40] Small G., Vorgan G. (2008). iBrain: Surviving the Technological Alteration of the Modern Mind.

[41] Carr N. (2010). The Shallows: What the Internet is Doing to Our Brains.

[42] Desmurget M. (2019). La Fabrique du Cretin Digital. Les Dangers des Ecranes Pour nos Enfants.

[43] Valkenburg P.M., Peter J. (2009). Social consequences of the Internet for adolescents: A decade of research. Curr. Dir. Psychol. Sci..

[44] Steinfield C.W. (1986). Computer-mediated communication in an organizational setting: Explaining task-related and socioemotional uses. Communication Yearbook 9.

[45] Hian L.B., Chuan S.L., Trevor T.M.K., Detenber B.H. (2004). Getting to know you: Exploring the development of relational intimacy in computer mediated communication. J. Comput.-Mediat. Commun..

[46] Hoffner C.A., Lee S. (2015). Mobile phone use, emotion regulation, and well-being. Cyberpsychol. Behav. Soc. Netw..

[47] Christopherson K.M. (2007). The positive and negative implications of anonymity in internet social interactions: “on the internet, nobody knows you’re a dog”. Comput. Hum. Behav..

[48] McKenna K.Y., Bargh J.A. (2000). Plan 9 from Cyberspace: The implications of the internet for personality and social psychology. Personal. Soc. Psychol. Rev..

[49] Deutsch R., Strack F. (2006). Target article: Duality Models in Social Psychology: From dual processes to interacting systems. Psychol. Inq..

[50] Kahneman D. (2011). Thinking, Fast and Slow.

[51] Zebrowitz L., Macrae C.N., Stangor C., Hewstone M. (1996). Physical Appearance as a Basis of Stereotyping. Stereotypes and Stereotyping.

[52] Asch S.E. (1946). Forming impressions of personality. J. Abnorm. Psychol..

[53] Livesley W.J., Bromley D.B. (1973). Person Perception in Childhood and Adolescence.

[54] Liggett J. (1974). The Human Face.

[55] Plichta P., Pyżalski J., Barlińska J. (2018). Cyberbullying Versus Self- Image Creation on the Internet: Are the Underlying Mechanisms Different in Young Adults with Disabilities?. Interdiscip. Contexts Spec. Pedagog..

[56] Menesini E., Nocentini A., Calussi P. (2011). The measurement of cyberbullying: Dimensional structure and relative item severity and discrimination. Cyberpsychol. Behav. Soc. Netw..

[57] Dehue F. (2013). Cyberbullying Research: New Perspectives and Alternative Methodologies. Introduction to the Special Issue. J. Community Appl. Soc. Psychol..

[58] Smith P.K., Mahdavi J., Carvalho M., Fisher S., Russell S., Tippett N. (2008). Cyberbullying: Its nature and impact in secondary school pupils. J. Child Psychol. Psychiatry.

[59] Völlink T., Bolman C.A.W., Dehue F., Jacobs N.C.L. (2013). Coping with cyberbullying: Differences between victims, bully-victims and children not involved in bullying. J. Community Appl. Soc. Psychol..

[60] Fonagy P., Twemlow S.W., Vernberg E.M., Nelson J.M., Dill E.J., Little T.D., Sargent J.A. (2009). A cluster randomized controlled trial of child- -focused psychiatric consultation and a school systems-focused intervention to reduce aggression. Child Psychol. Psychiatry.

[61] Thornberg R. (2007). A classmate in distress: Schoolchildren as bystanders and their reasons for how they act. Soc. Psychol. Educ..

[62] Endresen I.M., Olweus D., Bohart A., Stipek D. (2001). Self-reported empathy in Norwegian adolescents: Sex differences, age trends, and relationship to bullying. Constructive & Destructive Behavior: Implications for Family, School, & Society.

[63] Salmivalli C. (2010). Consequences of School Bullying and Violence. http://www.oecd.org/dataoecd/28/6/33866604.pdf.

[64] Machácková H., Dedkova L., Sevcikova A., Cerna A. (2013). Bystanders’ Support of Cyberbullied Schoolmates. J. Community Appl. Soc. Psychol..

[65] Creswell J.W., Poth C.N. (2017). Qualitative Inquiry & Research Design: Choosing among Five Approaches.

[66] Giménez-Gualdo A.-M., Arnaiz-Sánchez P., Cerezo-Ramírez F., Prodócimo E. (2018). Teachers’ and students’ perception about cyberbullying. intervention and coping strategies in primary and Secondary Education. Comunicar.

[67] Vandebosch H., Vandebosch H., Green L. (2019). Introduction. Narratives in Research and Interventions on Cyberbullying among Young People.

[68] Burr V. (2003). Social Constructionism.

[69] Castleberry A., Nolen A. (2018). Thematic analysis of qualitative research data: Is it as easy as it sounds?. Curr. Pharm. Teach. Learn..

[70] Van der Wal M.F., de Wit C.A., Hirasing R.A. (2003). Psychosocial health among young victims and offenders of direct and indirect bullying. Pediatrict.

[71] Macaulay P.J.R., Betts L.R., Stiller J., Kellezi B. (2021). ‘The more public it is, the more severe it is’: Teachers’ perceptions on the roles of publicity and severity in cyberbullying. Res. Pap. Educ..

[72] Elledge L.C., Cavell T.A., Ogle N.T., Newgent R.A. (2010). School-Based Mentoring as Selective Prevention for Bullied Children: A Preliminary Test. J. Prim. Prev..

[73] Ybarra M.L., Mitchell K.J. (2004). Online aggressor/targets, aggressors, and targets: A comparison of associated youth characteristics. J. Child Psychol. Psychiatry.

[74] Pyżalski J. (2012). Cyberbullying—Charakterystyka Zjawiska i Profilaktyka.

[75] Waasdorp T.E., Bradshaw C.P. (2015). The overlap between cyberbullying and traditional bullying. J. Adolesc. Health.

[76] Wójcik M., Kozak B. (2015). Bullying and exclusion from dominant peer group in Polish middle schools. Pol. Psychol. Bull..

[77] Menesini E., Codecasa E., Benelli B., Cowie H. (2003). Enhancing children’s responsibility to take action against bullying: Evaluation of a befriending intervention in Italian middle schools. Aggress. Behav..

[78] Kiesler S., Siegel J., McGuire T.W. (1984). Social Psychological Aspects of computer-mediated communication. Am. Psychol..

[79] Suler J. (2004). The online disinhibition effect. Cyberpsychol. Behav..

[80] Menesini E., Salmivalli C. (2017). Bullying in schools: The state of knowledge and effective interventions. Psychol. Health Med..

[81] Latané B., Darley J.M. (1970). The Unresponsive Bystander: Why Doesn’t he Help?.

[82] Ojala K., Nesdale D. (2004). Bullying and social identity: The effects of group norms and distinctiveness threat on attitudes towards bullying. Br. J. Dev. Psychol..

[83] Caravita S.C., Sijtsema J.J., Rambaran J.A., Gini G. (2014). Peer influences on moral disengagement in late childhood and early adolescence. J. Youth Adolesc..

[84] Mischel W. (2014). The Marshmallow Test: Mastering Self-Control.

[85] Hoffman M.L. (2000). Empathy and Moral Development.

[86] Szuster A., Jarymowicz M. (2020). Human empathy of automatic vs. reflective origin: Diverse attributes and Regulative Consequences. New Ideas Psychol..

[87] Cuff B.M.P., Brown S.J., Taylor L., Howat D.J. (2016). Empathy: A review of the concept. Emot. Rev..

[88] Preston S.D., de Waal F.B. (2000). Empathy: Its ultimate and proximate bases. Behav. Brain Sci..

[89] Decety J., Yoder K.J. (2016). Empathy and motivation for justice: Cognitive empathy and concern, but not emotional empathy, predict sensitivity to injustice for others. Soc. Neurosci..

[90] Rose C.A., Forber-Pratt A.J., Espelage D.L., Aragon S.R. (2013). The influence of psychosocial factors on bullying involvement of students with disabilities. Theory Into Pract..

[91] Pyżalski J. (2012). Agresja Elektroniczna i Cyberbullying Jako nowe Ryzykowne Zachowania Młodzieży.

[92] Kreuter M.W., Green M.C., Cappella J.N., Slater M.D., Wise M.E., Storey D., Clark E.M., O’Keefe D.J., Erwin D.O., Holmes K. (2007). Narrative communication in cancer prevention and control: A framework to guide research and Application. Ann. Behav. Med..

[93] Paul S., Smith P.K., Blumberg H.H. (2012). Comparing student perceptions of coping strategies and school interventions in managing bullying and cyberbullying incidents. Pastor. Care Educ..

[94] Cunningham C.E., Chen Y., Vaillancourt T., Rimas H., Deal K., Cunningham L.J., Ratcliffe J. (2015). Modeling the anti-cyberbullying preferences of University Students: Adaptive choice-based Conjoint Analysis. Aggress. Behav..

[95] Carrington S., Campbell M., Saggers B., Ashburner J., Vicig F., Dillon-Wallace J., Hwang Y.-S. (2017). Recommendations of school students with autism spectrum disorder and their parents in regard to bullying and cyberbullying prevention and intervention. Int. J. Incl. Educ..

[96] (2013). Cyberbullying and Children and Young People with SEN and Disabilities: The Views of Young People. SEN and Disability: Developing Effective Anti-Bullying Practice. Anti-Bullying Alliance. https://www.anti-bullyingalliance.org.uk/sites/default/files/field/attachment/disabled-young-peoples-views-on-cyberbullying-report.pdf.

[97] Maison D. (2019). Qualitative Marketing Research. Understanding Consumer Behaviour.

